# The GRE in Public Health Admissions: Barriers, Waivers, and Moving Forward

**DOI:** 10.3389/fpubh.2020.609599

**Published:** 2020-11-19

**Authors:** Jess A. Millar

**Affiliations:** ^1^Department of Epidemiology, University of Michigan, Ann Arbor, MI, United States; ^2^Department of Computational Medicine and Bioinformatics, University of Michigan, Ann Arbor, MI, United States

**Keywords:** GRE, waiver, graduate record examination, graduate applications, public health, SPH

## Abstract

In the wake of COVID-19, there is an urgent need for a diverse public health work force to address problems presented or exacerbated by the global pandemic. Educational programs that create our work force both train and shape the makeup of access through graduate applications. The Graduate Record Exam has a number of standing issues, with additional barriers created by the pandemic. We trace the GRE waiver movement over several years, focusing on the gradual adoption in CEPH accredited programs and the rapid expansion of temporary waivers as a response to testing access. Going forward, we need to consider gaps in waivers during the pandemic and how this data can be used to shape our future use of the GRE.

## Introduction

As we move forward in the profession of public health, many of the problems presented or exacerbated by the COVID-19 global pandemic may require new solutions and a diversity of thought and approaches to problem solving. The future of our public health workforce is shaped by decisions from our educational programs that decide who can access public health training and who designs the trainings. It is important that we consider how components of our graduate school applications currently shape our workforce and the possible barriers we create by the inclusion of testing metrics, such as the Graduate Record Exam (GRE). As the pandemic has unfolded, I have been active in collecting and sharing data on issues with the use of the GRE in public health admissions and I believe now is the time to re-think its problematic role in our public health workforce.

## Issues With the GRE

Over the last decade, issues with how the GRE increases barriers to graduate education have been more widely discussed. The GRE may not predict academic success, with correlation between GRE and academic success appearing to be weak at best (1). Boston University School of Public Health found no significant difference in mean GRE component scores for achieving >3.0 GPA in 1st year MPH students (2). The Association of Schools and Programs of Public Health also found no correlation between GRE scores and final GPA after public health degree completion at several of its member schools (3). Colorado SPH found GRE scores to be a weak predictor of degree completion, with other variables such as undergraduate GPA better predictors of success (4). And University of Minnesota conducted a randomized assessment, finding GRE score didn't substantially influence admissions decisions (5). Because of the financial burden and gender & racial/ethnic biases within the test, use of the GRE in public health admissions may create barriers for underrepresented groups. One of the direct barriers the GRE creates is a financial burden, with testing costing $205, and $27 per school submission. Another issue is the impact on diversity and inclusion efforts. Variation in scores by race and gender has been reported, with women and members of underrepresented racial and ethnic minority groups scoring lower than white and Asian men (6). Given that the GRE is not a convincing predictor of graduate school success, these barriers to entry are unnecessary.

The inequalities in testing have been increased during the COVID-19 pandemic. In March 2020 as lockdowns began and testing centers closed, ETS rolled out a solution to testing access: the GRE at Home. This version allowed testing to continue online, but came with a number of hurdles (7). The requirement of a desktop or laptop and stable internet connection to take the GRE at Home are particularly problematic, given the digital divide that has become more consequential during the pandemic. A 2019 Pew Research Center survey found one in four American adults lack access to high-speed internet. This increases to half for adults with an annual income <$30,000 in major US cities (8). In another study looking at undergrads at a large Midwestern university, although 98% of students had access to laptops, 20% of students still had difficulty accessing necessary education technology (9). These technology barriers create a further divide to accessing the test and shut out many students (7).

## GRE Waiver Movement

Recently, the practice of waiving the GRE in graduate applications has spread. In 2016, the American Astronomical Society recommended the elimination of the GRE due to the test's poor predictions of success, correlations with gender, race, and socioeconomic status, and financial burden (10). In November 2017 Joshua Hall director of the Biological & Biomedical Science Program at University of North Carolina at Chapel Hill, created a list of Bio/Biomedical programs that waive the GRE requirement (11). By the end of 2018, almost half of all top 50 ranked molecular biology programs had waived this requirement, with the practice spreading to more STEM disciplines (12). In 2019, some of the first high-ranked public health programs started to waive the GRE, including Boston University SPH and University of Colorado SPH (2, 4). In October 2019, a public health GRE waiver list of degrees/concentrations was created by Jess Millar, an Epidemiology MPH student at University of Michigan (13). At the time of its creation, 48 CEPH accredited programs (one in four) had at least one GRE waiver.

As COVID-19 started to spread in the United States and lockdowns were initiated, public health programs began to consider the possibility of temporarily waiving the GRE in light of barriers to the GRE at Home. By the beginning of April, Rutgers allowed temporary waivers for Fall 2020 (14). By the end of May, at least 9 CEPH accredited programs participated in the temporary waiver, with Emory extending their waivers to Fall 2021 (15, 16). The public health GRE waiver list increased 68% during its first 7 months, going from 145 to 243 entries by May 2020. By the time the SOPHAS application opened in August, the list had increased another 350% to include 880 entries (17). As of September 20th, 2020, the list contains 1,201 entries from 150 CEPH programs (just over three quarters). 560 of the entries are for concentrations/ degrees with a permanent GRE waiver, while 641 are temporary for COVID-19 (Supplementary Table).

## Public Health GRE Waiver Coverage Not Evenly Spread

**Figure 1:**
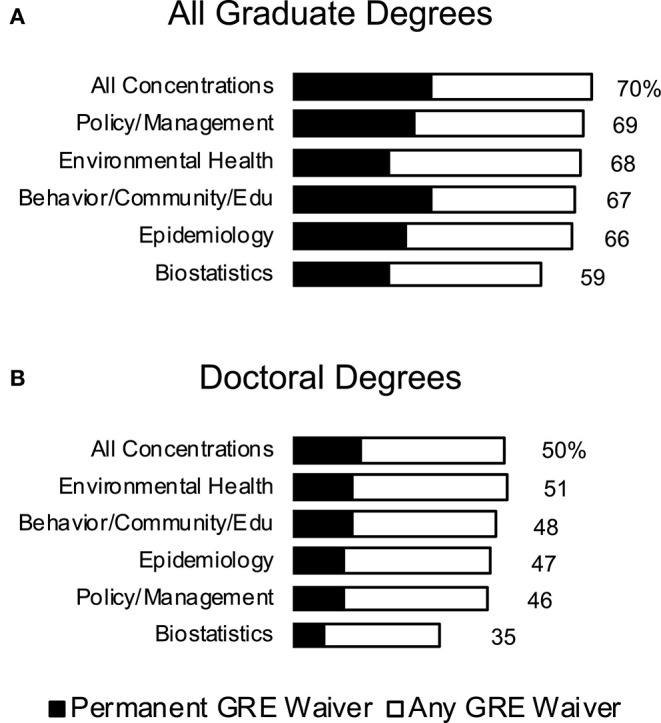
GRE Waiver Snapshot. Percent of degrees at CEPH accredited public health programs at U.S. universities that have a GRE waiver. All graduate degrees are shown in **(A)**, while only doctoral degrees are shown in **(B)**. Permanent GRE waivers are shown super imposed (black) over all GRE waivers (white). Percentage of degrees with any GRE waiver explicitly stated at the right of each bar. Not all degrees and concentrations were offered at each program.

The coverage of GRE waivers in public health programs is not equal, with very few programs allowing a blanket waiver to all graduate degrees. Among top 50 public health programs ranked by US World News, only 15 have a waiver for all degrees (permanent or temporary) as of September 20, 2020. Inclusion of a waiver also varies by concentration, with some programs including permanent waivers for specific degrees or concentrations, and temporary or no waivers for others. Seventy percentage of degrees in CEPH accredited programs currently offer at least a temporary GRE waiver, but that percent drops as low as 59% for admissions to biostatistics-specific degree programs (Figure 1A). These numbers drop to 33 and 23%, respectively, when only counting permanent waivers. The divide in GRE waivers is more apparent between masters and doctoral degrees. Among CEPH accredited programs, only 50% of degrees offer at least a temporary GRE waiver offered for doctoral degrees and 16% offer a permanent waiver (Figure 1B). Most doctoral programs require doctoral interviews for admission. These can and have been used by other disciplines—such as the aforementioned STEM programs–to devise interview questions to help identity characteristics found in successful doctoral researchers and rely less on the GRE (18).

## Conclusions

The pandemic has made inequities in access to education more visible, through the digital divide, financial concerns, and resulting conversations of barriers for minority groups. As we make our way into the 2020–2021 academic year, public health programs that have not done so may want to consider instigating or expanding temporary GRE waivers to more degrees and concentrations. Moving beyond the pandemic, there is a great deal more hesitancy on allowing more permanent GRE waivers. Several programs, such as Cornell, University of Iowa, and Ohio State, converted their temporary MPH GRE waivers to permanent (19, 20). University of Washington took it a step further, and converted their temporary GRE waivers to permanent for both masters and doctoral degrees (21).

Few studies on the GRE in public health programs have been conducted, but that is beginning to change. Boston University and University of North Carolina at Chapel Hill are currently conducting three-year studies to look at the impact of removing the GRE requirement on diversity and student success (2, 22). Several other programs are currently conducting 1-year pilot studies on the GRE waiver effect (23, 24). Temporary waivers are an opportunity for public health programs to test the relevance of GRE scores in the application process and their prediction of student success. We have the opportunity to test how removing a barrier to public health education will affect the professionals we create and I hope we take it.

## Data Availability Statement

The original contributions presented in the study are included in the article/Supplementary Material, further inquiries can be directed to the corresponding author.

## Author Contributions

JM drafted the initial manuscript, figures, edited, read, and approved the final manuscript.

## Conflict of Interest

The author declares that the research was conducted in the absence of any commercial or financial relationships that could be construed as a potential conflict of interest.

## Supplementary Material

Supplementary Table Table including all degrees/concentrations as of September 20, 2020 from CEPH accredited public health programs and whether they offer a GRE waiver.
